# Novel carbonyl iron-mediated synthesis of Pd–Fe magnetic nanomaterials: mechanism and catalytic performance in Suzuki coupling

**DOI:** 10.1098/rsos.260006

**Published:** 2026-10-21

**Authors:** Zhuangli Zhu, Jingwen Wang

**Affiliations:** ^1^ Science Education Centre, Tianjin Normal University, Tianjin, People's Republic of China; ^2^ Tianjin Hi-Tech Huayuan Foreign Languages School, Tianjin, People's Republic of China; ^3^ Faculty of Education, Tianjin Normal University, Tianjin, People's Republic of China

**Keywords:** Pd–Fe bimetallic nanomaterials, carbonyl iron-mediated synthesis, magnetic nanocatalysts, Suzuki–Miyaura coupling, sustainable catalysis

## Abstract

Palladium–iron (Pd–Fe) bimetallic catalysis represents a pivotal strategy for advancing the sustainability of organometallic transformations. Herein, we report a facile and efficient synthetic protocol for Pd–Fe magnetic nanomaterials mediated by carbonyl iron, which proceeds without external reducing agents, surfactants or protective gases. In this approach, carbonyl iron serves a dual role as both the iron source and the reducing agent, while *in situ* generated carbon monoxide provides three essential functions: atmospheric protection, pressure regulation and crystal nucleus stabilization. This unique reaction system facilitates the co-deposition of zero-valent palladium (Pd^0^) and iron oxide (Fe_2_O_3_) onto pristine Fe_3_O_4_ surfaces, yielding magnetically recyclable Pd–Fe@Fe_3_O_4_ nanocomposites. Comprehensive characterization via high-resolution transmission electron microscopy, scanning electron microscopy, energy-dispersive X-ray spectroscopy mapping and X-ray photoelectron spectroscopy confirms the well-defined hierarchical structure, uniform elemental distribution and controlled valence states of the as-synthesized materials. The resulting nanomaterials display a narrow particle size distribution (2–3 nm), high metal loading efficiency and low lattice mismatch. Preliminary catalytic evaluations reveal excellent activity of Pd–Fe@Fe_3_O_4_ in Suzuki–Miyaura cross-coupling reactions. Notably, similar performance is achieved with catalysts derived from other metal carbonyl precursors, underscoring the generality of this synthetic platform for designing efficient and recyclable bimetallic nanocatalysts.

## 1. Introduction

Palladium-catalysed carbon–carbon cross-coupling reactions are cornerstones of modern organic synthesis, with the Suzuki–Miyaura cross-coupling reaction being a representative example, owing to its mild reaction conditions and excellent functional group tolerance, a core achievement of the 2010 Nobel Prize in Chemistry [1]. In these research endeavours, the reduction of palladium dosage, enhancement of catalytic performance and investigation of reaction sustainability are of paramount importance. On the one hand, magnetic material-supported nanocomposite catalysts hold significant advantages in this regard [2–5]. On the other hand, the synergistic strategy of bimetallic catalysis can substantially improve catalytic efficiency and reduce the consumption of precious metals [6–10]. Notably, iron, the magnetic metal with the highest abundance in the Earth's crust, widest application and lowest cost, is an ideal candidate for synergistically catalysing the Suzuki reaction and reducing the usage of precious palladium [11].

Currently, the main preparation methods of magnetic material-supported palladium catalysts include one-pot aqueous synthesis [12], coprecipitation [13], polymer coating [14,15], surface functional modification [16–19], biosynthesis [20], impregnation reduction [21,22], core–shell structure design [23–25], supramolecular assembly [26,27], magnetic composite supports [28–30], and green synthesis strategies [4]. These methods have achieved high catalytic activity, easy recyclability and cyclic stability of the catalysts through the functional design of magnetic supports, precise palladium loading and structural optimization. However, most reported magnetic Pd-supported catalysts face inherent limitations: relying on specific ligands, linkers or complex precursors, alongside elaborate synthetic protocols. These issues not only increase fabrication complexity and cost but also impose unnecessary environmental and energy burdens. Such challenges hinder their industrial application, making the development of simpler, greener and more scalable catalytic systems highly valuable.

It is well established that iron carbonyl possesses reducibility, and its thermal decomposition constitutes an important method for the preparation of iron nanomaterials [31]. One of the core objectives of this work is to reduce its decomposition temperature and utilize its reducibility to *in situ* reduce palladium for the synthesis of Pd–Fe nanomaterials. Building on our previous work that the recycled products of iron carbonyl and metallic palladium can efficiently catalyse the Suzuki coupling reaction [11], herein we report a novel carbonyl iron-mediated synthesis of Pd–Fe magnetic nanomaterials. The as-prepared magnetic nanomaterials exhibit excellent activity for the model Suzuki coupling reaction of anisole-based substrates, as shown in figure 1. Based on literature support for metal carbonyl decomposition and our experimental phenomena, the main steps and proposed mechanisms are as follows. *Reduction stage*: pyrolysis of Fe(CO)_5_ and initiation of reduction; *nucleation stage*: *in situ* formation of Pd–Fe nanoparticles (NPs); *deposition stage*: directional deposition of Pd–Fe NPs onto Fe_3_O_4_. This method eliminates the need for additional reducing agents, surfactants or protective agents, directly employing carbonyl iron as both the iron source and reducing agent. It *in situ* generates carbon monoxide (CO) gas, which fulfils the functions of protection, pressure provision and crystal nucleus stabilization, yielding highly active and magnetically recyclable Pd–Fe@Fe_3_O_4_ catalysts (Pd nanoparticles strongly interacting with Fe_3_O_4_ support via electronic and structural synergy, with no bimetallic alloy formation).

**Figure 1.**
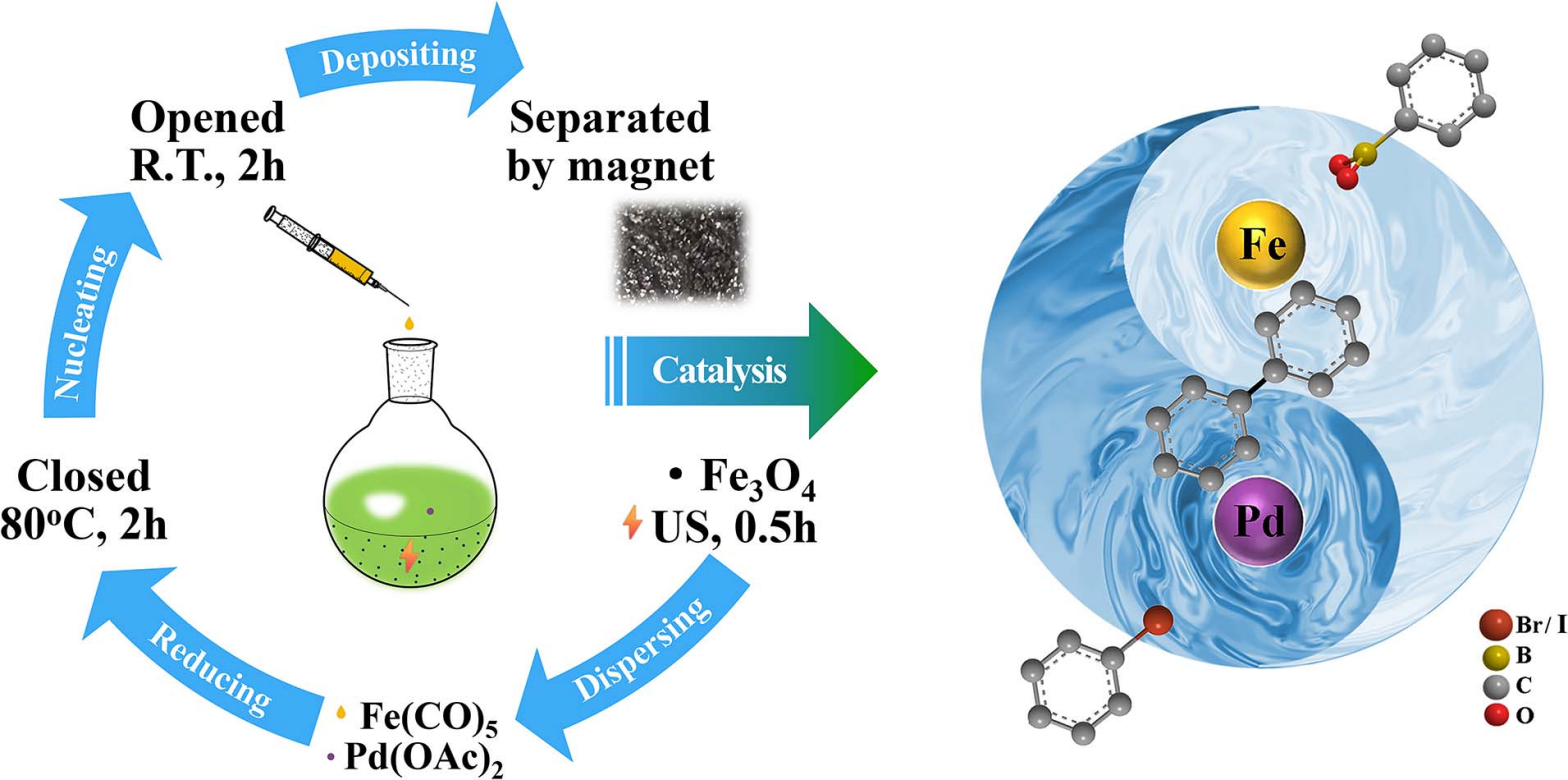
Mechanistic insight into carbonyl iron-mediated synthesis and Suzuki catalytic performance of Pd–Fe@Fe_3_O_4_.

## 2. Results and discussion

### 2.1. High-resolution transmission electron microscopy characterization and structure analysis

High-resolution transmission electron microscopy (HR-TEM) characterization revealed that the pristine magnetite (Fe_3_O_4_) powder used contains bulk, flake and rod-like microstructures (figure 2a). Pd–Fe NPs are distributed on the surface of these microstructures, and their particle sizes are mainly concentrated in the range of 2–3 nm (inset histogram in figure 2b). This particle size corresponds to the golden size for efficient catalytic coupling reactions (neither too large to reduce active sites nor too small to cause agglomeration) [32]. Analysis of random nanomaterial regions (figure 2b) showed that a large number of clear lattice fringes are visible (figure 2c). The lattice fringe with *d*_₁_ = 0.230 nm corresponds to the (1 1 1) plane of the Pd metal (PDF no. 46-1063); the lattice fringe with *d*_₂_ = 0.468 nm corresponds to the (1 1 1) plane of the Fe_3_O_4_ (PDF no. 26-1136).

**Figure 2.**
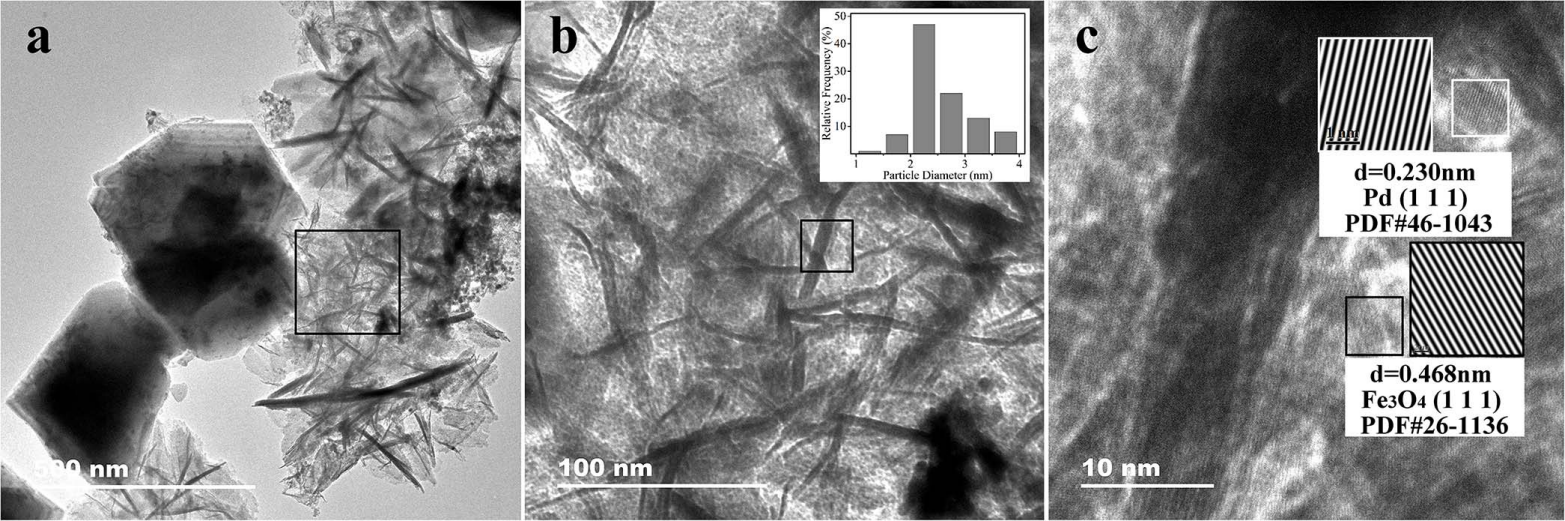
(a) TEM image of pristine Fe_3_O_4_ showing bulk, flaky and rod-like microstructures; (b) HR-TEM image of Pd–Fe@Fe_3_O_4_ with inset particle size distribution histogram; (c) lattice fringe analysis of Pd–Fe@Fe_3_O_4_ (Pd (1 1 1): *d* = 0.230 nm; Fe_3_O_4_ (1 1 1): *d* = 0.468 nm).

The calculated mismatch (Pd(1 1 1)/Fe_3_O_4_(1 1 1)) of 1.85% represents a 48.32% reduction compared with the standard value of 3.58% (supplementary material, table S3). This indicates remarkably high lattice matching quality, ultra-low interface strain energy, robust chemical bonds and excellent interface stability. The above results confirm that after palladium is reduced to the zero-valent state, it not only is firmly loaded on the surface of Fe_3_O_4_ but also optimizes and reconstructs the lattice through effects such as electron transfer, leading to lattice distortion and reduced lattice mismatch degree. Literature analysis shows that these effects include the electron transfer between palladium and iron [33] and the charge transfer between Fe^2+^ and Fe^3+^ induced by the deposition of zero-valent palladium [34]. The former promotes the electron transfer of (Pd^2+^ + Fe^2+^ → Pd^0^ + Fe^3+^) and improves surface adsorption mobility, while the latter increases the Fe^2+^/Fe^3+^ ratio and enhances stability.

### 2.2. Scanning electron microscopy characterization and morphology analysis

Scanning electron microscopy (SEM) characterization was also performed to complement the structure and morphology information. As presented in figure 3, the SEM images further confirm the multi-morphological features of the pristine Fe_3_O_4_ substrate, with bulk, flake and rod-like structures clearly observable. High-magnification SEM images demonstrate that the Pd–Fe NPs are uniformly anchored on the surface of the Fe_3_O_4_ microstructures without obvious agglomeration, which is consistent with the particle size distribution results obtained from HR-TEM characterization. This uniform dispersion of Pd–Fe NPs is favourable for exposing more active sites, thereby enhancing the catalytic performance of the composite material.

**Figure 3.**
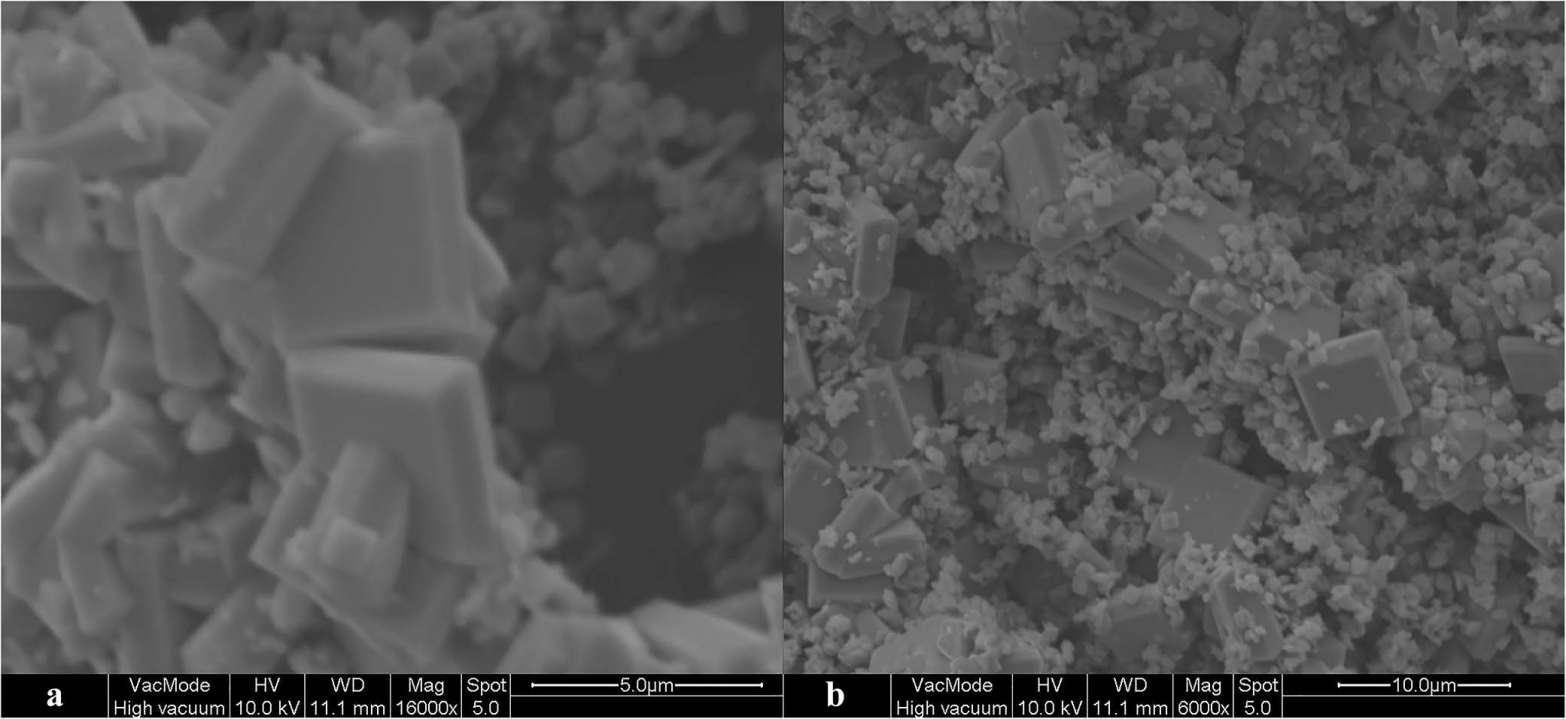
Enlarged views (a) and overall views (b) of blocky, flaky and rod-like microstructures of Pd–Fe@Fe_3_O_4_.

### 2.3. Energy-dispersive X-ray spectroscopy mapping and element distribution analysis

To determine the elemental distribution of the sample, energy-dispersive X-ray spectroscopy (EDS) mapping analysis was performed on a random region and the results are presented in figure 4. Based on the results of elemental distribution and error in figure 4, the Pd/Fe atomic ratio was calculated to be 0.045–0.049, which is consistent with the Pd/Fe atomic feeding ratio (0.045), indicating an extremely high palladium loading efficiency. Similarly, the Fe/O atomic ratio was calculated to be 0.710–0.711, slightly lower than the Fe/O atomic stoichiometric ratio in Fe_3_O_4_ (0.75), consistent with X-ray photoelectron spectroscopy (XPS) results (Fe^2+^/Fe^3+^ = 33.07%/66.93%), confirming the formation of Fe_2_O_3_. As illustrated in figure 4, the EDS mapping images clearly reveal the uniform distribution of Fe, O and Pd elements across the entire composite microstructure. The Fe element is widely distributed as the main component of the Fe_3_O_4_ substrate, while the O element distribution corresponds well to that of Fe, confirming the existence of Fe_3_O_4_. Notably, the Pd element exhibits a uniform and discrete distribution, which is consistent with the anchoring of Pd–Fe NPs on the Fe_3_O_4_ surface observed in HR-TEM and SEM characterizations. This uniform elemental distribution further verifies the successful formation of Pd–Fe@Fe_3_O_4_ and ensures the uniform exposure of active Pd sites, which is crucial for improving the catalytic activity and stability.

**Figure 4.**
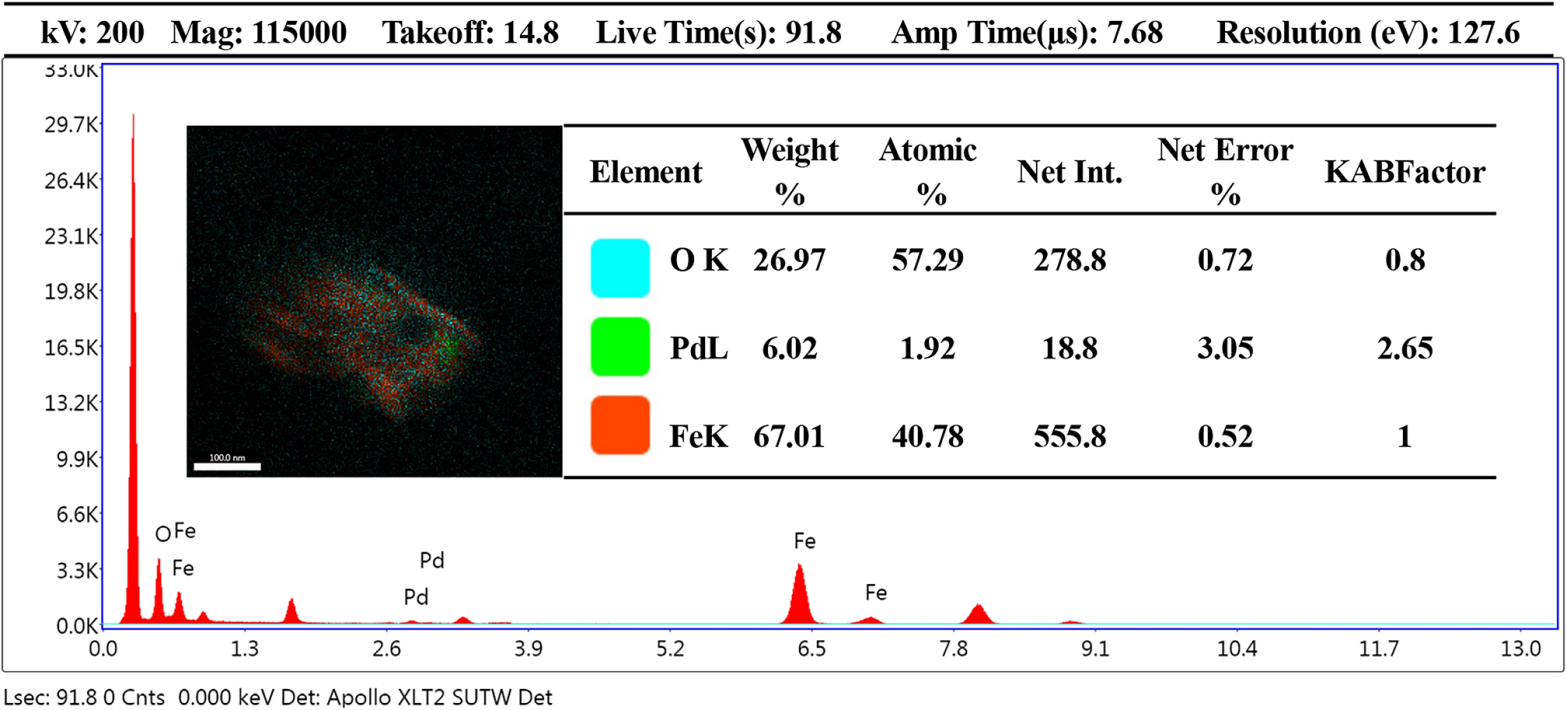
Element distribution in a random region.

### 2.4. X-ray photoelectron spectroscopy and element valence state analysis

XPS analysis was conducted to investigate the valence states of Pd and Fe elements in the composite. As shown in figure 5, the results confirmed that palladium was reduced to the zero-valent state, while iron existed in the forms of Fe^2+^ and Fe^3+^. The XPS spectrum of the Fe 2p peak (figure 5a) showed a peak separation (Δ*E* = 13.6 eV). Utilizing the peak positions of Fe^2+^ at 709.5 eV and Fe^3+^ at 710.9 eV, the Fe 2p peak could be deconvoluted into two fitted peaks. The relative area ratio of the deconvoluted peaks attributed to Fe^2+^ and Fe^3+^ was 33.07% : 66.93%, which was slightly lower than the atomic stoichiometric ratio of Fe^2+^ to Fe^3+^ in Fe₃O₄ (33.3% : 66.7%) [35]. Combined with the results of electron microscopy and elemental analysis, this ratio difference indicated that a small amount of Fe_2_O_3_ was introduced into the system. The XPS spectrum of the Pd 3d peak (figure 5b) showed a peak separation (Δ*E* = 5.3 eV). The binding energies of Pd 3d_5/2_ and Pd 3d_3/2_ were 334.8 and 340.1 eV, respectively, indicating that palladium was in the zero-valent state.

**Figure 5.**
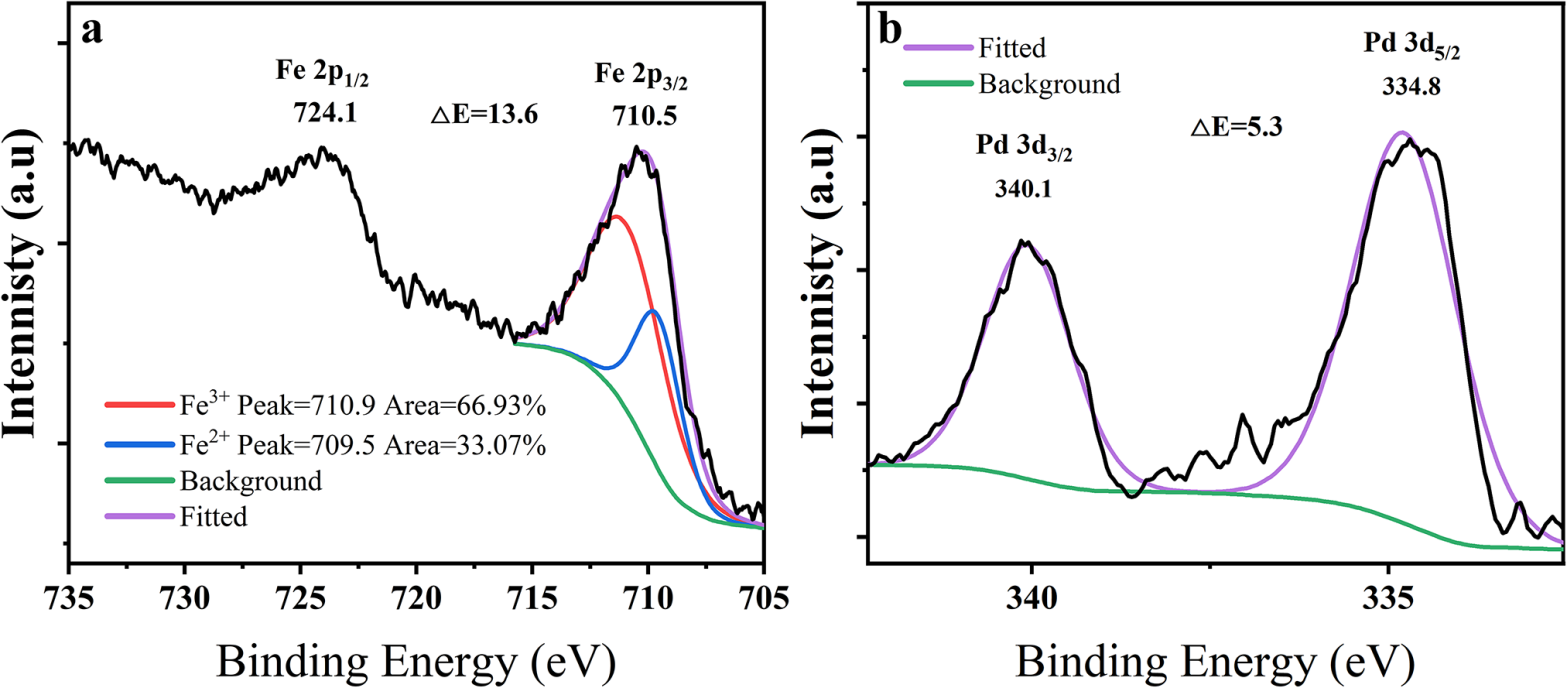
XPS spectra of the Fe 2p peaks (a) and the Pd 3d peaks (b).

The XPS analysis revealed distinct binding energy shifts of Pd (Pd^0^ 3d_5/2_: 334.8 eV, a 0.3 eV lower shift than bulk Pd^0^ (335.1 eV)) and Fe species (Fe^3+^ 2p_3/2_: 710.9 eV, a 0.1 eV lower shift than that of pure Fe_3_O_4_ (711.0 eV)) in the Pd NPs@Fe_3_O_4_ system, reflecting the strong metal–support interaction between Pd NPs and the Fe_3_O_4_ support [15,34]. Specifically, the lower Pd 3d_5/2_ binding energy was attributed to the electron donation to Pd^0^ from Fe^2+^ and oxygen vacancy sites on Fe_3_O_4_. Correspondingly, the slightly lower Fe^3+^ 2p_3/2_ binding energy of Fe_3_O_4_ was due to the reverse electron feedback from Pd^0^ to Fe^3+^. This bidirectional electron transfer between Pd and Fe_3_O_4_ optimized the electronic structure of the catalyst, which is favourable for the catalytic performance in Suzuki–Miyaura cross-coupling reactions [23].

### 2.5. Catalytic performance analysis

Catalytic performance analysis was systematically conducted to evaluate the activity and stability of Pd–Fe@Fe_3_O_4_ in the Suzuki–Miyaura cross-coupling reaction (table 1). As shown in table 1, the reactions with bromo- (entry 3) and iodo-substituted anisoles (entry 4) achieved excellent yields of 90% and 99%, respectively, accompanied by high turnover number (TON) values of 371 and 408. This reactivity trend (I > Br) is consistent with the inherent activity order of aryl halides in Suzuki–Miyaura coupling reactions, where C–I bonds are more easily activated than C–Br bonds. In contrast, the reactions with fluoro- (entry 1) and chloro-substituted anisoles (entry 2) failed to yield detectable products, which can be attributed to the high bond dissociation energy of C–F and C–Cl bonds that are difficult to cleave under the current reaction conditions. It was found that successful couplings require the presence of Pd–Fe@Fe_3_O_4_, as determined by the control reactions in the preliminary experiment (supplementary material, scheme S1), where only the reaction catalysed by the prepared Pd–Fe@Fe_3_O_4_ gave 85% yield, while reactions that we attempted using equal amounts of Pd(OAc)_2_, Fe_3_O_4_ or Pd(OAc)_2_ and Fe_3_O_4_ did not lead to product formation.

**Table 1.** Suzuki catalytic performance of Pd–Fe@Fe_3_O_4_.

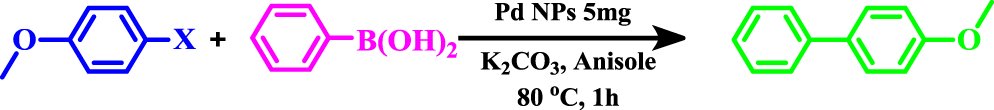				
entry	X	yield^*a*^	turnover number	catalytic cycle^*b*^
1	F	—	—	—
2	Cl	—	—	—
3	Br	90 ± 1.73%	371	—
4	I	99 ± 1.00%	408	1
5	I	94 ± 2.00%	387	2
6	I	89 ± 1.00%	367	3
7	I	88 ± 2.65%	363	4
8	I	85 ± 1.73%	350	5
9	I	83 ± 2.00%	342	6

^*a*^Reaction conditions: X–Ar 0.5 mmol, PhB(OH)_2_ 0.5 mmol, Pd–Fe@Fe_3_O_4_ 5.00 mg, [Pd] 2424 ppm calculated by the actual amount of Pd used, K_2_CO_3_ 1 mmol, anisole 3 ml, 80°C. Yield (± standard deviation (*n* = 3)) was determined by purification.

^*b*^After each reaction, we placed a magnet at the bottom of the reaction flask, poured off the supernatant, washed the catalyst residue with ethyl acetate and ethanol and then put it into the next cycle.

Furthermore, the recycling performance of the Pd–Fe@Fe_3_O_4_ catalyst was investigated using iodo-substituted anisole as the model substrate (entries 4–9). After each reaction cycle, the catalyst was efficiently recovered via magnetic separation, followed by washing with ethyl acetate and ethanol to remove residual reactants and products, and then directly reused in the next cycle without additional activation. As indicated by the data in table 1, the catalyst maintained good catalytic efficacy over five successive recycling cycles (entries 5–9; figure 6).

**Figure 6.**
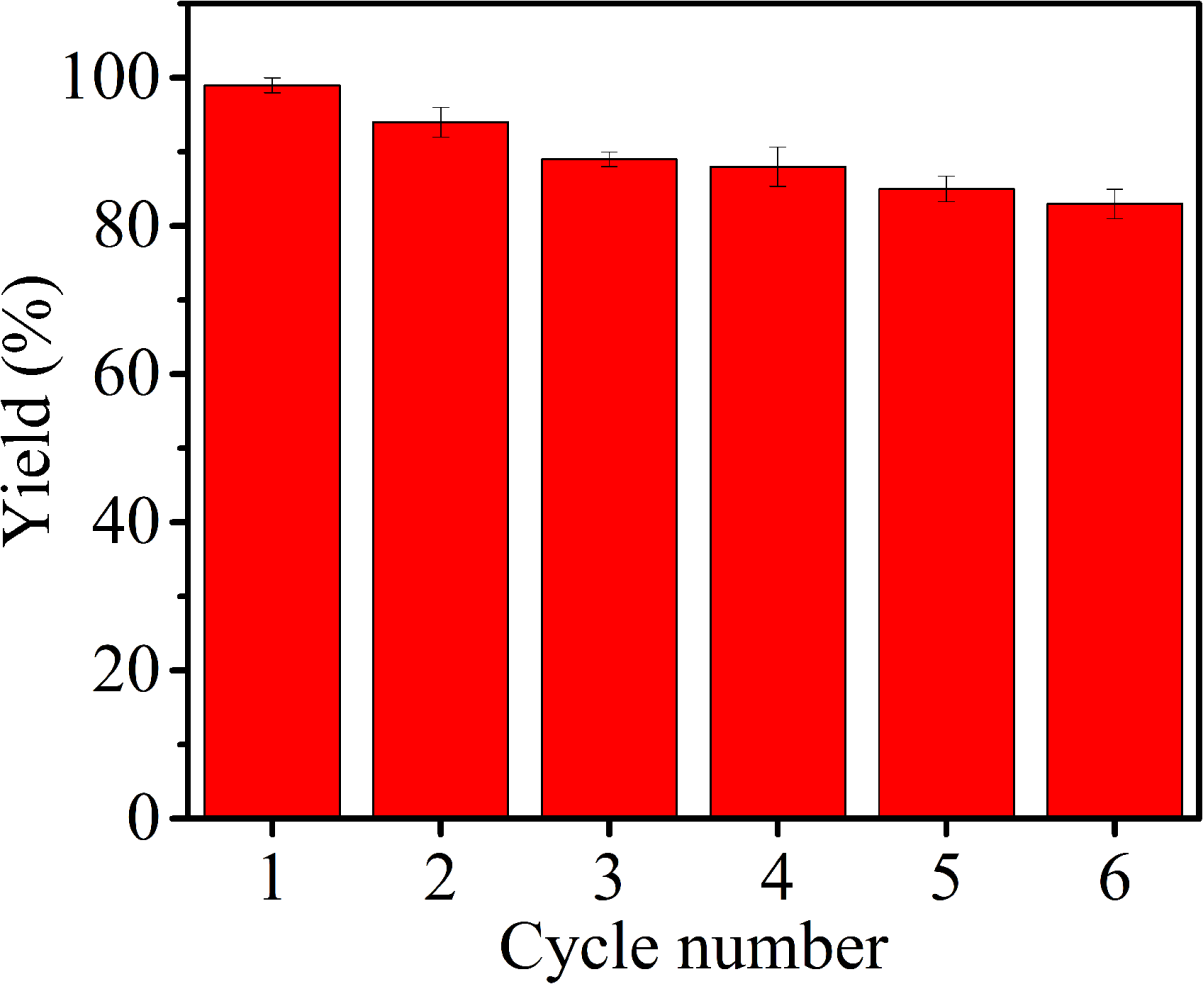
Suzuki catalytic cycle of Pd–Fe@Fe_3_O_4_ (error bars: standard deviation (*n* = 3)).

The product yield slightly decreased from 99% (first cycle, entry 4) to 83% (sixth cycle, entry 9), and the corresponding TON values declined from 408 to 342. Even after six cycles, the yield remained above 80%, demonstrating satisfactory cycling stability. Post-catalysis characterization (HR-TEM and XPS) revealed no significant agglomeration of Pd–Fe NPs, which confirmed that the robust chemical bonding between Pd–Fe NPs and the Fe_3_O_4_ substrate (verified by HR-TEM lattice matching analysis) and the uniform dispersion of active Pd sites (confirmed by EDS mapping) effectively suppressed the leaching and agglomeration of active components during the recycling process.

### 2.6. Extension of metal carbonyl-mediated synthetic approaches

To extend the applicability of this synthetic method, other metal carbonyl compounds, namely Mo(CO)_6_, Cr(CO)_6_, Fe_3_(CO)_12_ and Fe_2_(CO)_9_, were used to replace Fe(CO)_5_. A series of magnetic Pd NPs were prepared via the same method as previously described, and their catalytic performance was evaluated using the identical Suzuki reaction model and reaction conditions (the coupling reaction between 4-iodo-substituted anisole and phenylboronic acid). It was found that all these magnetic Pd NPs catalytic systems achieved excellent catalytic effects, as summarized in table 2.

**Table 2.** Suzuki catalytic performance of magnetic Pd NPs.

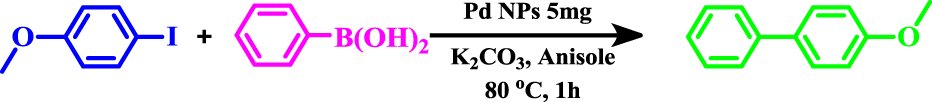				
metal carbonyl	Fe_3_(CO)_12_	Fe_2_(CO)_9_	Mo(CO)_6_	Cr(CO)_6_
isolated yield	90%	77%	98%	86%

For the series of iron carbonyl compounds employed (Fe(CO)_5_, Fe_3_(CO)_12_ and Fe_2_(CO)_9_), a clear correlation was observed between their solubility and the resulting catalytic performance: as their solubility decreased (Fe(CO)_5_ > Fe_3_(CO)_12_ > Fe_2_(CO)_9_), the catalytic yields of the corresponding magnetic Pd NPs catalysts also decreased, respectively (99%, 90% and 77%). This positive correlation between solubility and catalytic yield was primarily ascribed to enhanced solubility of iron carbonyl compounds in the reaction medium (anisole), which promoted their uniform dispersion and adequate interaction with the palladium precursors. Meanwhile, Fe_2_(CO)_9_ exhibited extremely poor stability at 80°C and decomposed within several minutes; this not only significantly increased the difficulty associated with the preparation of magnetic Pd NPs but also led to a marked deficiency in the catalytic yield. Precisely considering its lower thermal decomposition temperature, better solubility in common organic solvents, relatively single decomposition products and a more controllable decomposition rate, we selected Fe(CO)_5_ as the core precursor instead of Fe_3_(CO)_12_ and Fe₂(CO)_9_.

Mo(CO)_6_, Cr(CO)_6_ and Fe_3_(CO)_12_ exhibited comparable solubility, while the corresponding magnetic Pd NPs catalysts showed satisfactory catalytic performance. Among them, Cr(CO)_6_ possessed relatively high stability, which retarded the formation rate of magnetic Pd NPs and slightly lowered its catalytic yield. In contrast, the magnetic Pd NPs prepared from Mo(CO)_6_ afforded significantly superior catalytic performance, which was attributed to the similarities in atomic structure and chemical properties between Mo and Pd, as well as their higher affinity in catalytic synergy.

## 3. Experimental

### 3.1. General methods and materials

All reagents were purchased from commercial suppliers and used without further purification unless specified otherwise. Flash chromatography was performed using 200–300 mesh silica gel with the indicated solvent system according to standard techniques and analytical thin-layer chromatography was carried out using 250 mm commercial silica gel plates. ^1^H NMR and ^13^C NMR spectra were recorded on a Bruker AVANCE III 400 MHz (400 MHz for ^1^H; 101 MHz for ^13^C) spectrometer in CDCl_3_. For ^1^H NMR, tetramethylsilane (TMS) served as an internal standard (δ = 0) and ^1^H NMR chemical shifts are reported in ppm downfield of TMS and referenced to the residual solvent peak (CDCl_3_ at 7.26 ppm) unless otherwise noted. The data are reported as follows: chemical shift, integration, multiplicity (s = singlet, d = doublet, t = triplet, q = quartet, m = multiplet), and coupling constant in hertz. For ^13^C NMR, CDCl_3_ was used as an internal standard (δ = 77.16) and spectra were obtained with complete proton decoupling.

### 3.2. Safety statement

Metal carbonyl compounds including Fe(CO)_5_, Fe_3_(CO)_12_, Fe₂(CO)_9_, Mo(CO)_6_ and Cr(CO)_6_ are toxic and volatile substances that may cause serious harm to human health. All operations must be carried out in a well-ventilated fume hood. Proper personal protection (laboratory coat, safety goggles and nitrile gloves) is required during the whole experiment. These reagents should be stored in sealed containers in a cool and dry place away from heat and incompatible materials. All procedures strictly follow the relevant safety regulations to ensure the safety and reproducibility of experiments. Detailed information on the metal carbonyl compounds is as follows:

— *Iron pentacarbonyl (Fe(CO)_5_)*. Purity: ≥98.0%. Storage: sealed in a dry, cool and dark place, away from heat, flames and oxidants. Safety: highly toxic and volatile; all operations must be performed in a fume hood.
— *Tri-iron dodecacarbonyl (Fe_3_(CO)_12_)*. Purity: ≥97.0%. Storage: sealed and stored in a cool, dry and well-ventilated environment. Safety: toxic and moisture-sensitive; avoid inhalation and skin contact.
— *Di-iron nonacarbonyl (Fe_2_(CO)_9_)*. Purity: ≥97.0%. Storage: kept sealed in a cool and dark place, protected from light and heat. Safety: harmful if inhaled or touched; handle only in a fume hood.
— *Molybdenum hexacarbonyl (Mo(CO)_6_)*. Purity: ≥98.0%. Storage: sealed in a dry and cool cabinet, away from incompatible substances. Safety: toxic and thermally sensitive; may release toxic CO upon heating.
— *Chromium hexacarbonyl (Cr(CO)_6_)*. Purity: ≥98.0%. Storage: stored in a sealed container under cool and dry conditions. Safety: highly toxic; may cause serious damage to organs; use only in a fume hood.

### 3.3. Catalyst characterization

The morphologies of samples were observed by HR-TEM images, using a JEOL 100 CXII transmission electron microscope working at 200 kV. The elemental mapping information of the samples was characterized by EDS mapping analysis, carried out on a TEM-EDAX TEAM system working at 200 kV (resolution of 127.6 eV). The larger-scale morphologies of samples were observed by SEM images, using a JEOL JSM-7600F scanning electron microscope working at 10 kV as the acceleration voltage. The elemental valence information of samples was characterized by XPS, carried out on a VG ESCALAB 250 XPS system with a monochromatized Al Kα X-ray source working at 15 kV and 200 W.

### 3.4. Catalyst preparation method

First, 100 mg of iron(II, III) oxide (Fe_3_O_4_) was thoroughly dispersed in 5 ml of anisole in a 10 ml Schlenk tube, followed by ultrasonic agitation for 30 minutes, where the selection of solvent was a critical factor for success. Second, 0.17 mmol (21 µl) of iron pentacarbonyl (Fe(CO)_5_) and 0.027 mmol (0.0061 g) of palladium(II) acetate (Pd(OAc)_2_) were added. The tube was sealed and heated to 80°C via temperature-programmed heating, and then stirred for 2 hours. Subsequently, the reaction system was stirred under open conditions for another 2 hours. Finally, the product was collected using a magnet, washed successively with ethyl acetate and ethanol, and dried under vacuum to obtain a black magnetic powder (110 mg). For the preparation of magnetic catalytic materials using other metal carbonyl compounds, only Fe(CO)_5_ needs to be replaced with the corresponding metal carbonyl compounds, while the reaction steps and reactant molar ratios remain unchanged.

### 3.5. Experimental method for the Suzuki cross-coupling reaction

The reactants and Pd–Fe@Fe_3_O_4_ catalyst were placed in a 10 ml Schlenk tube equipped with a magnetic stir bar. Then 3 ml of anisole was added, the tube was sealed tightly, and the mixture was stirred and heated at 80°C for 1 hour. After cooling to room temperature, the mixture was extracted with ethyl acetate two to three times. The combined extracts were dried over anhydrous sodium sulfate and purified by silica gel column chromatography using 200–300 mesh silica gel with petroleum ether/ethyl acetate = 15:1 (v/v) as the eluent. The reaction process and product purity were monitored by thin-layer chromatography using commercial 250 µm silica gel plates with petroleum ether/ethyl acetate = 20:1 (v/v) as the developing solvent, and the spots were visualized under UV light (254 nm). The target product, 4-methoxybiphenyl, was obtained finally, with its spectral data as follows: ^1^H NMR (400 MHz, CDCl_3_) δ (ppm): 7.42 (t, *J* = 9.1 Hz, 4H), 7.29 (t, *J* = 7.5 Hz, 2H), 7.18 (t, *J* = 7.3 Hz, 1H), 6.85 (d, *J* = 8.3 Hz, 2H), 3.70 (s, 3H).^13^C NMR (101 MHz, CDCl_3_) δ (ppm): 159.3, 140.9, 133.9, 128.8, 128.2, 126.8, 126.7, 114.3, 55.4.

### 3.6. Catalyst recovery and reuse method

After each reaction, a magnet was placed at the bottom of the Schlenk tube, and the reaction solution was decanted. The residual solid in the tube was washed sequentially with ethyl acetate, water and ethanol, which was retained as the recycled catalyst. The cycling tests were then carried out following the above-mentioned experimental method for the Suzuki cross-coupling reaction.

### 3.7. Calculation method for Pd dosage

Based on the total amount of Pd(OAc)_2_ used in catalyst preparation, the Pd dosage for each reaction was calculated according to the ratio of the catalyst mass per reaction to the total mass of the prepared catalyst. The formula is shown as follows:

$$\begin{array}{r} Pd\;(mol\%)=\frac{n\;\left(Pd{\left(OAc\right)}_{2}\right)\times \frac{m\;\left(catalyst\;per\;reaction\right)}{m_{0}\;\left(total\;catalyst\;prepared\right)}}{n\;\left(aryl\;halide\right)}\;\times 100. \end{array}$$

## 4. Conclusions

In summary, a novel method for carbonyl iron-mediated synthesis of Pd–Fe@Fe_3_O_4_ magnetic catalysts, integrating iron source, reducing agent and *in situ* CO protection in a single step through the reduction stage, nucleation stage and deposition stage. The catalysts feature a narrow particle size distribution (2–3 nm), high Pd loading efficiency, low lattice mismatch (1.85%) and excellent catalytic performance (up to 99% isolated yield) and recyclability (83% yield after six cycles) in Suzuki coupling reactions. This strategy is extendable to other metal carbonyl compounds (e.g. Mo(CO)_6_, Cr(CO)_6_), providing a versatile platform for designing low-cost, recyclable bimetallic catalysts. Future work will focus on detailed mechanistic studies on CO's precise role, the systematic optimization of reaction conditions, extension of substrate scope, comprehensive comparison and sustainability for industrial scalability.

## Supplementary Material



## Data Availability

All information regarding experimental instruments, reagents and methods has been provided in the paper and supplementary material [36]. No other data, code or other digital research materials were involved.
