# Supplementary material for: Novel carbonyl iron-mediated synthesis of Pd–Fe magnetic nanomaterials: mechanism and catalytic performance in Suzuki coupling
Source: R Soc Open Sci. 2026 Oct 21;13(10):260006. doi: 10.1098/rsos.260006 (PMC13682864; doi:10.1098/rsos.260006)
Supplement: Supplementary file 1 [file rsos.260006.zip › rsos.260006/rsos260006_si_001.docx]

**Electronic Supporting Maderias for**

Novel Carbonyl Iron-Mediated Synthesis of Pd-Fe Magnetic Nanomaterials: Mechanism and Catalytic Performance in Suzuki Coupling

Zhuangli Zhu,^*^*^a^* Jingwen Wang*^b^* and Qiushi Su^a^

*^a.^*	Science Education Centre, Tianjin Normal University, No.393 West Binshui Road, Tianjin, China. Email: zlzhu@tjnu.edu.cn

*^b.^*	Tianjin Hi-Tech Huayuan Foreign Languages School, No.5 Haitai Fazhan 1st Road, Tianjin, China.

c.	Faculty of Education, Tianjin Normal University, No.393 West Binshui Road, Tianjin, China.

**List of Contents**

1. Solvent screening experiments in the catalyst preparation---------------S1

2. Calculation of Pd% loading--------------------------------------------------S1

3. Lattice Mismatch Calculation Method-------------------------------------S2

4. Controlled catalytic reactions and Pd leaching test-----------------------S2

5. XPS details (C and O) of Fe-Pd@Fe_3_O_4_------------------------------------S3

6. Characterization of 4-methoxy-1,1'-biphenyl-----------------------------S3

7. Copies of ^1^H-NMR and ^13^C-NMR Spectra---------------------------------S4

Detailed experimental and characterization methods are provided in the main text. Additional supporting data are included below.

**1.** **Solvent screening experiments in the catalyst preparation**

Table S1 Solvent screening experiments in the catalyst preparation

| Entry | Solvent | Support | Pd NPs | Yield/%*^a^* |
| --- | --- | --- | --- | --- |
| 1 | PhCH_3_ | Fe_3_O_4_ | 5mg | 90 |
| 2 | CH_3_CN | Fe_3_O_4_ | 5mg | 70 |
| 3 | H_2_O | Fe_3_O_4_ | 5mg | 16 |
| 4 | EtOH | Fe_3_O_4_ | 5mg | 28 |
| 5 | PhOCH_3_ | Fe_3_O_4_ | 5mg | 91 |

*^a^*Reaction conditions: Ar-X (0.5mmol), Ar’-B(OH)_2_ (0.5mmol), Pd-Fe@Fe_3_O_4_ (5mg, [Pd] 2424ppm), K_2_CO_3_ (1mmol), EtOH/H_2_O(3mL, v/v = 1:1), ultrasound at ambient temperature, Yield was determined by GC and confirmed by purification. Unless otherwise stated, the Suzuki coupling reactions were performed under the aforementioned conditions.

Experimental results show that the yield of the Suzuki reaction exhibits a rising trend with increasing boiling points of these polar solvents, except for water, while the boiling points of solvents follow the order: PhOCH₃ > PhCH₃ > CH₃CN > EtOH > H₂O. Experiments show that Fe(CO)_5_ is easily decomposed by heat in water to form yellow Fe_2_(CO)_9_, thereby blocking the formation of Pd–Fe NPs, resulting in a low yield of the Suzuki cross coupling reaction.

**2. Calculation of Pd% loading**

The Pd loading was determined based on the actual amount of Pd utilized in the preparation of the catalyst, rather than the nominal loading with the following formula:

$$Pd mol\%=\frac{n\left( {\mathrm{Pd}\left( \mathrm{OAc} \right)}_{2} \right)*\frac{m\left( catalyst per reaction \right)}{m_{0}\left( total catalyst prepared \right)}}{n\left( aryl halide \right)}*100\%$$

$$=\frac{0.027mmol*\frac{5mg}{110mg}}{0.5mmol}*100\%$$

=2424 ppm

**3. Lattice Mismatch Calculation Method**

Lattice parameters (Table S2) of the as-prepared sample can be derived from the lattice fringe spacing observed in HRTEM images and the corresponding (hkl) indices using the following formula:

$$a=d\times\sqrt{h^{2}+k^{2}+l^{2}}$$

Table S2 Lattice parameters of the samples

| Parameters/nm | Pd-Fe@Fe₃O₄ | Reference(PDF) |
| --- | --- | --- |
| *a_1_*/Pd(111) | 0.398 | 0.390 (#46-1043) |
| *a_2_*/Fe₃O₄(111) | 0.811 | 0.809 (#26-1136) |

Table S3 Comparison of lattice mismatch degrees

| Mismatch%*^a^* | Pd-Fe@ Fe₃O₄ | standard value | Relative Reduction% |
| --- | --- | --- | --- |
| Pd(111)/Fe₃O₄(111) | 1.85 | 3.58 | 48.32 |

*^a^*For mismatch of Pd(111)/Fe₃O₄(111), The lattice parameter 2a₁ of Pd(111) corresponds to a₂ of Fe₃O₄(111). Mismatch [Pd(111)/Fe₃O₄(111)] % = ${|{2a}_{1}-a_{2}|}/{a_{2}}\times100\%$

**4. Controlled catalytic reactions and Pd leaching test**

It was found that successful couplings require the presence of Pd-Fe@Fe_3_O_4_, as determined by the control reactions in preliminary experiment in Scheme S1, where only reaction catalyzed by prepared Pd-Fe@Fe_3_O_4_ (60 ^o^C, 0.66mg/320ppm [Pd]) gave 85% yield while reactions that we attempted using equal amounts of Pd(OAc)_2_, Fe_3_O_4_, or Pd(OAc)_2_ and Fe_3_O_4_ did not lead to product formation (Scheme S1a-d).

To confirm the heterogeneous nature of the catalysis and evaluate the stability of the catalyst, a mercury poisoning test and Pd leaching analysis were performed. In the mercury poisoning experiment (Scheme S1e), the addition of Hg(0) resulted in an almost complete suppression of catalytic activity, indicating that the active sites are Pd⁰ nanoparticles on the catalyst surface and that the reaction proceeds via a heterogeneous pathway. Inductively coupled plasma atomic emission spectrometry (ICP-AES, Prodigy, Leeman Labs) analysis was performed to evaluate metal leaching, while Pd was not detected (ND) in the reaction filtrate.

Scheme S1 Control reactions proved the importance of Pd-Fe@Fe_3_O_4_

**5. XPS details(C and O) of Fe-Pd@Fe_3_O_4_**

**6. Characterization of 4-methoxy-1,1'-biphenyl.**

**4-methoxy-1,1'-biphenyl. ^1^H NMR (400 MHz, CDCl_3_) δ (ppm): 7.42 (t, J = 9.1 Hz, 4H), 7.29 (t, J = 7.5 Hz, 2H), 7.18 (t, J = 7.3 Hz, 1H), 6.85 (d, J = 8.3 Hz, 2H), 3.70 (s, 3H). ^13^C NMR (101 MHz, CDCl_3_) δ (ppm): 159.3, 140.9, 133.9, 128.8, 128.2, 126.8, 126.7, 114.3, 55.4.**

**7. Copies of ^1^H-NMR and ^13^C-NMR Spectra.**

**^1^H NMR (400 MHz) and ^13^C{^1^H} NMR (101 MHz) spectra of 4-methoxy-1,1'-biphenyl (CDCl_3_).**
